# The effect of social suspicion on social media addiction among Chinese college students: A moderated mediation model

**DOI:** 10.1371/journal.pone.0323474

**Published:** 2025-05-21

**Authors:** Tao Wei, Xiao-Li Xing, Jing-Jing Liu, Yan Gan, Xue Gong, Xiu-Fang Zhang, Bu Xu, Xiang-Xia Rong

**Affiliations:** The First Affiliated Hospital of Anhui University of Science and Technology, Huainan, Anhui, China; İstanbul Nişantaşı Üniversitesi: Istanbul Nisantasi Universitesi, TÜRKIYE

## Abstract

**Objective:**

This study investigates the impact of social suspicion on social media addiction among Chinese college students, examining the mediating role of the sense of meaning in life and the moderating effect of gender.

**Methods:**

A cross-sectional survey was conducted from September 19, 2023, to November 2, 2023, involving 1,558 students from three universities in southern Anhui Province, China. Participants completed questionnaires assessing social suspicion, social media addiction, and the sense of meaning in life. Data were analyzed using correlation analysis, Hayes’ PROCESS macro, and bootstrap methods to test mediation and moderation effects.

**Results:**

The findings revealed a bidirectional relationship between social suspicion and social media addiction. Social suspicion positively predicted social media addiction, with the sense of meaning in life mediating this relationship. Gender moderated the mediating effect, as the indirect effect of social suspicion on social media addiction through the sense of meaning in life was significantly stronger among female students compared to male students. Additionally, social media addiction also positively predicted social suspicion, with the sense of meaning in life and gender moderating this relationship.

**Conclusion:**

This study reveals a bidirectional relationship between social suspicion and social media addiction among Chinese college students, mediated by a sense of meaning in life and moderated by gender. Social suspicion directly drives addiction while indirectly exacerbating it through reduced the sense of meaning in life, with females showing stronger mediation effects and males exhibiting heightened susceptibility to suspicion when addicted. These findings emphasize the need for gender-tailored interventions to address psychological vulnerabilities and mitigate risks of digital overuse.

## Introduction

In the digital age, social media platforms have become an indispensable facet of daily life, particularly among college students, who are often early adopters of emerging technologies. These platforms offer unparalleled opportunities for communication, self-expression, and information sharing, yet they also pose significant risks, including the potential for social media addiction. Characterized by compulsive usage and detrimental effects on mental health, academic performance, and interpersonal relationships, social media addiction has emerged as a pressing public health concern [1–3]. While extensive research has identified various predictors of social media addiction, the role of social suspicion, a psychological construct reflecting mistrust in social interactions, remains underexplored. In recent years, research on the psychological mechanisms of social media addiction has intensified, with scholars uncovering its formative dynamics through multidimensional analyses of personality traits, emotional needs, and cognitive patterns. Extensive empirical studies have identified neuroticism [4], loneliness [5], and fear of missing out [6] as significant predictors of excessive use behaviors. From a social cognitive perspective, research further highlights the critical role of online social compensation mechanisms in addiction development. Individuals with social skill deficits or real-world social anxiety often turn to virtual interactions, substantially increasing their risk of social media dependency [7]. Notably, while trust—a foundational element of social interaction—has been examined in social media use [8,9], the impact of social suspicion on addictive behaviors remains underexplored. This theoretical gap limits our holistic understanding of the cognitive-affective interplay in social media dependency. This limitation is particularly salient given the profound ways in which social media has reshaped social dynamics, creating both opportunities for connection and challenges related to overuse. College students, who are navigating the dual pressures of academic and social life, represent a population particularly susceptible to these risks, making them a critical focus for understanding the mechanisms underlying social media addiction.

The vulnerability of college students to social media addiction is further underscored by their developmental stage and social context. As they transition to adulthood, college students often face heightened demands for social integration and self-identity formation, which may drive excessive reliance on social media platforms [10,11]. According to compensatory internet use theory [12], individuals with social suspicion may over-rely on social media to compensate for mistrust in offline interactions, thereby increasing addiction risk. For example, those perceiving offline interactions as untrustworthy may seek solace in the perceived control and anonymity of social media, reinforcing compulsive usage [7,13].

The sense of meaning in life, a psychological construct reflecting an individual’s perception of purpose and value in their existence, plays a pivotal role in mitigating maladaptive behaviors, including social media addiction. Grounded in self-determination theory [14], the sense of meaning in life is closely tied to intrinsic motivation and psychological well-being, serving as a protective factor against excessive reliance on external validation. Research indicates that individuals with a strong sense of meaning are less likely to engage in compensatory behaviors, such as excessive social media use, as they derive fulfillment from intrinsic sources rather than external platforms [14,15]. Conversely, individuals who lack a sense of purpose may turn to social media as a substitute for existential fulfillment, seeking validation and connection in virtual spaces to compensate for deficiencies in real-life interactions [6,16]. This compensatory mechanism is particularly relevant in the context of social suspicion, as individuals who mistrust real-world social interactions may disproportionately rely on social media to fulfill unmet psychological needs. Thus, the sense of meaning in life is hypothesized to mediate the relationship between social suspicion and social media addiction, offering a pathway through which interpersonal mistrust translates into compulsive digital behaviors.

Gender further complicates this dynamic, as research indicates distinct patterns of social media use and addiction risk between males and females. For instance, females are more likely to use social media for relational purposes, such as sharing personal information or maintaining friendships, which may heighten their vulnerability to addiction [17,18]. These gender differences suggest that the pathways linking social suspicion, a sense of meaning in life, and social media addiction may vary significantly across genders, warranting a nuanced exploration. Moreover, emerging evidence suggests that social media addiction itself may exacerbate feelings of social suspicion, particularly among individuals who experience a lack of a sense of meaning in life or who are highly dependent on social media for emotional validation [19]. This bidirectional relationship underscores the need to examine not only how social suspicion predicts addiction but also how addiction may reinforce social suspicion over time.

Based on the theoretical frameworks and empirical evidence discussed above, we propose the following hypotheses:

H1: Social suspicion will positively predict social media addiction among college students.
H2: The sense of meaning in life will mediate the relationship between social suspicion and social media addiction.
H3: Gender will moderate the mediating effect of the sense of meaning in life on the relationship between social suspicion and social media addiction.
H4: Social media addiction will also predict higher levels of social suspicion, with a sense of meaning in life and gender moderating this relationship.

## Materials and methods

### Participants and procedure

We conducted a cross-sectional survey among students in southern Anhui Province from September 19 to November 2, 2023. Using random sampling, we selected multiple classes from each grade. Data collection was carried out through online questionnaires, with links distributed via WeChat groups. To ensure data integrity, our system restricted participation to one questionnaire submission per IP address.

The study protocol received ethical approval from the Research Ethics Committee of the First Affiliated Hospital of Anhui University of Science and Technology. Following ethical guidelines and considering the study’s minimal risk profile, the committee granted a waiver of informed consent for all participants, including both adult respondents and guardians of minors.

In the initial sample of 1914 participants, we implemented rigorous quality control measures. Responses completed in less than 200 seconds, which was our predetermined threshold for adequate response time, were excluded from the analysis. This quality assurance process resulted in the exclusion of 356 responses, yielding a final analytical sample of 1558 valid questionnaires that met our predefined quality criteria.

### Research tools

#### Social suspicion scales.

The Social Suspicion Scale, which was developed by Linett and colleagues in 2019, has excellent psychometric qualities and may be used by both clinical populations (particularly patients who suffer from social anxiety) and non-clinical groups (such as college students and members of the community) [20]. Every item describes the degree to which individuals are mistrustful and suspicious of others. Additionally, these entries may be sensitive to detecting changes in social suspicion in patients who suffer from social anxiety before and after therapy. The Chinese version of the Social Suspicion Scale was used in this study [21]. Scholars in China tested the Social Suspicion Scale extensively to ensure its validity and reliability after adapting it to the country’s cultural context. This examination was administered to a group of college students, proving that it can be used to reliably and accurately measure social suspicion in populations outside of clinical settings. The Chinese iteration of the Social Suspicion Scale comprises 18 inquiries, which correspond to a score spectrum ranging from 0 (indicating complete non-compliance) to 4 (indicating complete compliance). Higher scores on this scale signify a stronger inclination to perceive the conduct of others in social contexts in a negative or hostile manner, as well as a diminished level of trust in others. Confirmatory factor analysis indicated good construct validity (χ^2^/df = 1.978, CFI = 0.937, TLI = 0.915, SRMR = 0.058, and RMSEA = 0.025). The internal consistency of the scale was assessed using Cronbach’s alpha and KMO test coefficients (Bartlett’s test, *P* < 0.05). The obtained values were 0.924 and 0.953, respectively, suggesting that the scale demonstrates satisfactory levels of reliability and validity.

#### Sense of meaning in life scale.

Some of the existing instruments for gauging the significance of life include the following: the personal meaning profile created by researchers in Taiwan [22], the Sense of Meaning in Life Scale by Steger et al. [23], and The Purpose in Life Test by Crumbaugh and Maholick [24], along with other updated versions of the aforementioned instruments. Unfortunately, the measurement capabilities of these scales are limited, and they are 20 years old. There are significant cultural variations, as well as a change in spatial and temporal context, for Chinese college students due to the country’s present fast social growth. Taking all of this into consideration, a Chinese scholar named Jinlong Liang [25] reworked the College Student Meaning in Life Scale for university students in a way that is more in line with Chinese cultural norms, drawing on Frankl’s theory of meaning therapy and making reference to Wu et al.’s Sense of life Meaning Scale. The scale comprises 13 questions that assess three factors: freedom of will, will to seek meaning and meaning of life. Confirmatory factor analysis indicated good construct validity (χ^2^/df = 1.821, CFI = 0.971, TLI = 0.964, SRMR = 0.016, and RMSEA = 0.070). The scale’s internal consistency was evaluated by calculating Cronbach’s alpha and KMO test coefficients. Bartlett’s test was used to determine statistical significance (*P* < 0.05). The obtained scores of 0.894 and 0.951 indicate that the scale exhibits satisfactory levels of reliability and validity.

#### Social media addiction scale.

One way to measure addiction is via the Social Media Addiction Scale [26]. On the six dimensions of salience, craving, emotion control, relapse, withdrawal, and conflict, the Chinese version of the Social Media Addiction Scale presents eighteen items. There are three questions in each dimension, and the possible responses range from 1 (never) to 5 (always). An individual’s level of addiction is proportional to their total score. Confirmatory factor analysis indicated good construct validity (χ^2^/df = 1.872, CFI = 0.937, TLI = 0.915, SRMR = 0.058, and RMSEA = 0.051). According to Bartlett’s test (*P* < 0.05), the scale’s KMO test coefficient was 0.907 and the internal consistency Cronbach’s alpha was 0.919, suggesting that the scale is valid and reliable.

### Statistical analysis

The statistical analysis of the study was conducted using SPSS 23.0 software. Data were presented as the mean value with its standard deviation (Mean ± SD). Additionally, Pearson’s correlation analysis was utilized to explore the correlation between variables at a significance level of α = 0.05. Harman’s one-factor test was used to ascertain the presence of common method bias in the research. Confirmatory factor analysis, performed using Mplus 8, was employed to assess the validity of the variables. Model 4 in Hayes’ (2022) PROCESS macro was used in a mediation analysis to examine the mediating role of college students’ sense of meaning in life. Moreover, we conducted an examination of moderated mediation by using Model 8 as an essential component of the PROCESS macro to ascertain if sex influenced either the indirect or direct pathway.

## Results

### General demographic data of subjects

This study included 1558 people, 765 of whom were male (49.1% of the total) and 793 of whom were female (50.9%). Regarding the age distribution, 1097 respondents (70.41%) were aged ≤18, while 461 respondents (29.59%) were aged 19–21. Detailed demographic information is summarized in Table 1.

**Table 1:** General demographic data of subjects (n = 1558).

	Variables	Number	Percentage(%)
Total		1558	100
Gender	Male	765	49.10
	Female	793	50.90
Age	<18	1097	70.41
	18-21	455	29.59
Residence	City	306	80.36
	Town	1252	19.64

### The test of common method bias

To evaluate the standard procedure, the Harman single-factor test [27] was used. A total of 13 non-rotating components were identified by the exploratory factor analysis. The variance interpretation percentage of the first principal component was less than 40% (32.26%), suggesting that the measurement did not exhibit any major common method bias.

### Confirmatory factor analysis

Before hypothesis testing, the measurement model was rigorously evaluated using confirmatory factor analysis (CFA) to ensure construct validity. The model incorporated three latent factors: social suspicion, social media addiction, and the sense of meaning in life. As detailed in Table 2, the three-factor model demonstrated a strong fit to the empirical data, with fit indices well within acceptable thresholds [χ²(1124) = 1847.856, CFI = 0.924, TLI = 0.911, SRMR = 0.048, RMSEA = 0.039]. These results not only confirm the model’s robustness but also highlight its superiority over competing factor structures. Importantly, the CFA findings provide compelling support for the discriminant validity of the measurement tools, underscoring the distinctiveness and theoretical independence of the constructs under investigation.

**Table 2:** Results of confirmatory factor analysis.

Models	Variables	χ²	df	χ²/df	CFI	TLI	SRMR	RMSEA
Three-factor model	Social Suspicion, sense of meaning in life, Social Media Addiction	1,847.856	1,124	1.644	0.924	0.911	0.048	0.039
Two-factor model	Social Suspicion+sense of meaning in life, Social Media Addiction	2,265.512	1,126	2.012	0.851	0.831	0.059	0.047
Single-factor model	Social Suspicion+sense of meaning in life+Social Media Addiction	2,681.133	1,127	2.379	0.731	0.727	0.059	0.056

### Demographic comparisons of social suspicion, sense of meaning in life, and social media addiction

There were significant differences in social suspicion, sense of meaning in life, and social media addiction scores among different gender, age, and residence groups (Table 3). Specifically, social suspicion scores varied significantly by gender, with females reporting higher levels than males (*t* = 5.020, *P* < 0.001). Sense of meaning in life scores differed significantly by age, with individuals aged 19–21 reporting higher scores than those aged 18 and younger (*t* = 2.078, *P* < 0.05), and by residence, with city residents reporting higher scores than town residents (*t* = 2.273, *P* < 0.05). No significant differences were observed in social media addiction scores across gender, age, or residence groups. These findings highlight the varying influences of demographic factors on psychological and behavioral outcomes.

**Table 3:** Comparisons of Social Suspicion, Social Media Addiction, and the Sense of Meaning in Life scores among different demographic characteristics.

	Variables	Number	Social Suspicion	Sense of Meaning in Life	Social Media Addiction
Gender	Male	765	15.16 ± 4.96	47.95 ± 11.10	38.05 ± 11.64
	Female	793	16.54 ± 5.85	47.90 ± 11.34	37.62 ± 11.62
*t*			5.020	0.097	0.718
*P value*			<0.001	0.992	0.473
Age	≤18	1,097	16.13 ± 5.99	47.03 ± 11.31	37.88 ± 11.04
	19-21	455	15.73 ± 5.16	48.33 ± 11.11	37.85 ± 11.87
*t*			1.250	2.078	0.039
*P value*			0.211	<0.05	0.968
Residence	City	306	15.39 ± 5.73	49.23 ± 11.32	37.88 ± 13.01
	Town	1,252	15.94 ± 4.92	47.61 ± 11.17	37.83 ± 11.27
*t*			1.690	2.273	0.048
*P value*			0.091	<0.05	0.962

### Correlation analysis of social suspicion, social media addiction, and the sense of meaning of life respectively

Local correlation analyses were conducted for variables controlling for age and place of residence to exclude interference from demographic variables. Table 4 shows the mean, standard deviation, and correlation values for all observed variables. In addition, social suspicion was positively correlated with social media addiction and negatively correlated with the sense of meaning in life, while gender with social suspicion was weakly negatively correlated.

**Table 4:** Correlation analysis of Social Suspicion, Social Media Addiction, and the Sense of Meaning in Life respectively.

	(Scores)M	SD	1	2	3	4
1 Sex^a^	0.51	0.50	1.000			
2 Social Suspicion	15.195	5.844	-0.054^*^	1.000		
3 Sense of meaning in life	47.924	11.219	0.002	-0.173^***^	1.000	
4 Social Media Addiction	37.838	11.629	0.018	0.450^***^	-0.023	1.000

*P < 0.05, ***P < 0.001.

^a^ Sex is a dummy variable, Male = 0, Female = 1.

### Testing for the mediation model

All variables were standardized before analysis. Mediation pathways were examined using PROCESS V4.0 Macro (Model 8), with age, gender, and residence included as covariates. A bias-corrected bootstrap analysis with 5,000 resamples was performed to construct 95% confidence intervals (CI) for effect estimation.

Social suspicion significantly predicted both a sense of meaning in life (*β* = −0.175, *P* < 0.001) and social media addiction (*β* = 0.459, *P* < 0.001) (Table 5). The construct exhibited a robust negative association with a sense of meaning in life (*β* = −0.175, *P* < 0.001) alongside a pronounced positive association with social media addiction (*β *= 0.459, *P* < 0.001). Notably, a sense of meaning in life independently predicted increased social media addiction (*β *= 0.053, *P* = 0.020), indicating that heightened perceptions of meaning correlate with elevated addictive behaviors.

**Table 5:** The mediation analysis of Sense of Meaning in Life between Social Suspicion and Social Media Addiction.

Predictors	Model1(Sense of meaning in life)					95% confidence interval		Model2(Social Media Addiction)					95% confidence interval	
	*B*	*β*	*SE*	*t*	*P* *value*	BootLLCI	BootULCI	*B*	*β*	*SE*	*t*	*P value*	BootLLCI	BootULCI
Residence	-1.452	-0.051	0.705	-2.059	0.040	-2.835	-0.069	-0.144	-0.005	0.662	-0.217	0.828	-1.443	1.155
Gender	-0.146	-0.007	0.561	-0.261	0.794	-1.246	0.954	0.973	0.042	0.526	1.850	0.065	-0.059	2.004
Age	-0.475	-0.051	0.233	-2.038	0.042	-0.931	-0.018	-0.640	-0.066	0.219	-2.929	0.003	-1.069	-0.211
Social suspicion	-0.153	-0.175	0.022	-7.008	0.000	-0.194	-0.110	0.416	0.459	0.021	20.000	0.000	0.375	0.457
Sense of Meaning in Life								0.059	0.053	0.024	2.328	0.020	0.009	0.102
*R^2^*	0.030							0.205						
*F*	48.218							201.045						

The mediation analysis revealed a suppression effect, where the direct (*β* = 0.460, 95% CI [0.415, 0.505]) and indirect (*β* = −0.010, 95% CI [−0.021, −0.001]) effects operated in opposing directions (Table 6). Specifically, social suspicion directly increased addiction but indirectly reduced it through diminished a sense of meaning in life (accounting for -2.222% of the total effect). The net positive total effect confirms the predominance of the direct pathway.

**Table 6:** The 95% Confidence Interval of the mediating effect test and deviation corrections.

Trails	Effect value	BootSE	95% confidence interval		Effect ratio
			BootLLCI	BootULCI	
Total effect	0.450	0.023	0.405	0.494	
Social Suspicion → Social Media Addiction	0.460	0.023	0.415	0.505	102.222%
Social Suspicion → Sense of meaning in life → Social Media Addiction	-0.010	0.005	-0.021	-0.001	-2.222%

Among covariates, age demonstrated consistent negative associations with both a sense of meaning in life (*β* = −0.051, *P* = 0.042) and social media addiction (*β* = −0.066, *P* = 0.003). However, none of the residential variables reached statistical significance (*P* > 0.05) in any of the pathways, suggesting minimal confounding effects of these demographic factors.

### Sensitivity analysis of the mediation model

To rigorously evaluate the robustness of the mediation effect, we conducted a comprehensive sensitivity analysis encompassing three primary approaches: (1) outlier exclusion, (2) systematic manipulation of control variables, and (3) gender-stratified subgroup analyses. Detailed information regarding the analytical framework is presented in Table 7. The full sample analysis revealed a statistically significant mediation effect of a sense of meaning in life between social suspicion and social media addiction (*β* = -0.010, 95% CI [-0.021, -0.001]), indicating that social suspicion influences social media addiction partially through reduced sense of meaning in life.

**Table 7:** Robustness Checks for Mediation Effects Across Analytical Specifications.

Analysis Type	Sample Size	Total Effect	Direct Effect	Mediation Effect	95% Confidence Interval (BootLLCI, BootULCI)
Full Sample Analysis	1,558	0.450	0.460	-0.010	(-0.021, -0.001)
Excluding Outliers	1,500	0.445	0.455	-0.009	(-0.020, -0.001)
Introducing Controls	1,558	0.448	0.458	-0.010	(-0.021, -0.001)
Excluding Controls	1,558	0.452	0.462	-0.010	(-0.021, -0.001)
Gender Subgroup (Male)	765	0.440	0.450	-0.009	(-0.020, -0.001)
Gender Subgroup (Female)	793	0.460	0.470	-0.011	(-0.022, -0.001)

The robustness of this finding was confirmed through multiple sensitivity tests. First, the exclusion of outliers (defined as observations beyond ±3 standard deviations from the mean) yielded consistent results (*β* = -0.009, 95% CI [-0.020, -0.001]), demonstrating that extreme values did not unduly influence the mediation effect. Second, systematic manipulation of control variables, including the addition of demographic covariates (age, gender, and residence) and their subsequent removal, produced stable effect estimates (*β* = -0.010, 95% CI [-0.021, -0.001]), indicating minimal sensitivity to covariate specification.

Gender-stratified analyses revealed nuanced patterns in the mediation pathway. While the effect size was marginally smaller in males (*β* = -0.009, 95% CI [-0.020, -0.001]) compared to females (*β* = -0.011, 95% CI [-0.022, -0.001]), the mediation effect remained statistically significant in both subgroups, suggesting consistent pathway activation across genders.

These comprehensive sensitivity analyses collectively demonstrate the stability and reliability of the identified mediation effect. The consistent pattern of results across multiple analytical conditions provides strong empirical support for the proposed model, wherein social suspicion exerts an indirect influence on social media addiction through the mediating mechanism of a reduced sense of meaning in life.

### Testing for moderated mediation and reverse causality

**Fig 1:**
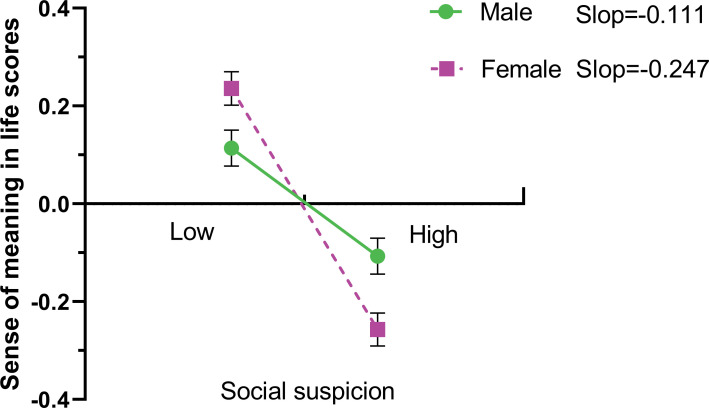
The simple slope test of the moderator effect of gender on the relationship between social suspicion and the sense of meaning in life of college students.

The findings showed that the interaction of social suspicion with gender had a significant effect on the sense of meaning in life (*β* = -0.136,  *P*< 0.05), but unfortunately not on social media addiction (Table 8). These results suggest that gender may have influenced the association between social suspicion, the sense of meaning in life, and social media addiction. In addition, we performed simple slope analyses to account for these significant interactions and to explore whether male slopes differed from female slopes in this model. Fig 1 shows the relationship between social suspicion and the sense of meaning in life by gender. Specifically, social suspicion was found to have a larger negative influence on the sense of meaning in life in women (*β*_simple_ = -0.247, *t* = 6.734,  *P*< 0.05) than in males (*β*_simple_ = -0.111, t = 3.242, *P* < 0.05).

**Table 8:** Regression results of moderated mediation.

Predictors	Sense of meaning in life			Social Media Addiction		
	*β*	*t*	*P* value	*β*	*t*	*P* value
Social Suspicion	-0.111	-3.242	0.001	0.465	14.980	0.000
Sense of meaning in life				0.057	2.465	0.014
Gender	-0.014	-2.825	0.006	0.086	1.893	0.058
Social Suspicion*Gender	-0.136	-2.721	0.007	-0.006	-0.124	0.902
R^2^	0.035			0.207		
F	18.622			101.526		

**Fig 2:**
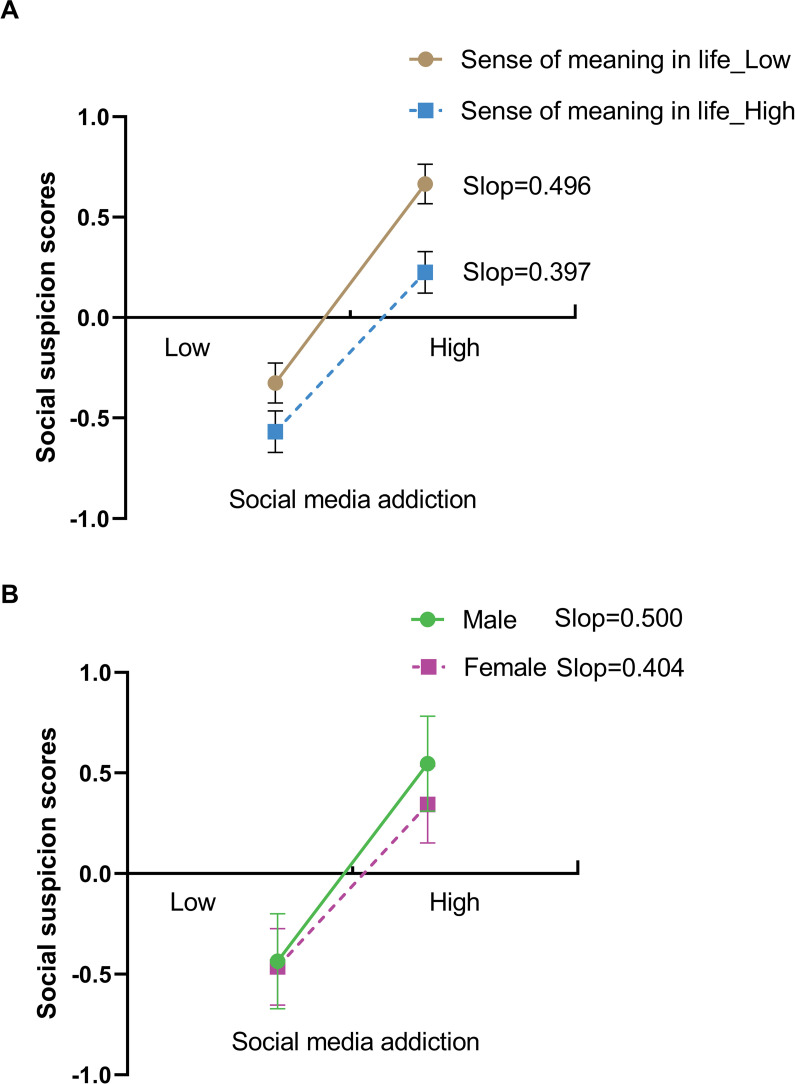
Differential Predictive Effects of Social Media Addiction on Social Suspicion: Roles of Gender and Sense of Meaning in Life.

To probe directional complexity, we tested a reverse causal model (PROCESS Model 2) with social media addiction as the independent variable, social suspicion as the outcome, and gender and the sense of meaning in life as moderators. Results demonstrated significant moderating effects for both the sense of meaning in life (Fig 2A) and gender (Fig 2B). Escalating social media addiction predicted heightened social suspicion more prominently in males (*β*_simple_ = 0.500, 95% CI [0.432, 0.568]) than in females (*β*_simple_ = 0.404, 95% CI [0.336, 0.472]). Concurrently, the relationship between social media addiction and social suspicion varied by individuals’ sense of meaning in life: the association was stronger among those with low sense of meaning in life (*β*_simple_ = 0.496, 95% CI [0.416, 0.576]) compared to those with high sense of meaning in life (*β*_simple_ = 0.397, 95% CI [0.317, 0.477]), indicating that diminished meaning perception amplified the addictive behavior-suspicion link.

## Discussion

This study uncovers a multifaceted relationship between social suspicion, social media addiction, and the sense of meaning in life, with several critical findings that extend current understandings of digital behavior. Notably, the results reveal a bidirectional dynamic: while social suspicion directly predicts higher social media addiction, social media addiction itself also exacerbates social suspicion, particularly among males and individuals with a low sense of meaning in life. Additionally, gender and sense of meaning in life emerge as significant moderators, shaping the strength and direction of these associations.

### Bidirectional relationships between social suspicion and social media addiction

This study elucidates the multifaceted mechanisms through which social suspicion influences social media addiction, revealing a complex psychodynamic model comprising both direct and mediated pathways. Structural equation modeling analysis demonstrates that social suspicion not only directly predicts social media addiction but also establishes an indirect pathway mediated by the sense of meaning in life. At the direct effect level, our findings confirm Hypothesis 1, indicating that cognitive vigilance in social interactions may drive compensatory digital engagement to address deficiencies in offline social trust - a mechanism empirically supported by multiple studies [17,28]. When individuals maintain skeptical attitudes toward real-world social relationships, they demonstrate a propensity to seek alternative emotional connections through social media platforms, a behavioral pattern particularly pronounced in adolescent populations. Empirical evidence demonstrates that adolescents experiencing peer rejection frequently resort to high-frequency social media interactions as an emotional compensation mechanism due to eroded real-world social trust (i.e., heightened social suspicion), significantly elevating their addiction vulnerability [29]. Moreover, existing evidence suggests that negative emotional states (e.g., anxiety, and loneliness) constitute critical precursors to social media addiction. Crucially, social suspicion may intensify these psychological distress responses, thereby indirectly reinforcing addictive behaviors [30], further arguing for our findings. Notably, persistent doubts about others’ intentions may compel individuals to engage in compulsive social media validation-seeking behaviors to confirm self-worth or obtain psychological security. These findings advance our understanding of the psychopathological mechanisms underlying digital addiction and provide empirical evidence for developing targeted intervention strategies addressing maladaptive coping patterns in interpersonal relationships.

Moreover, the study reveals that social media addiction is a positive predictor of social suspicion, which further argues for Hypothesis 4. These findings are consistent with existing evidence suggesting that excessive social media users are more likely to fall prey to overinterpretations of others’ intentions and trust deficits, which may stem from the constant occupancy of cognitive resources by platform design and anxiety induced by social comparison [29,31]. For example, frequent viewing of carefully curated content (e.g., friend circles or updates) may trigger “social surveillance” behaviors, whereby users’ self-doubt or questioning of others’ motives through side-by-side comparisons reinforces suspicious tendencies [32,33]. Research further suggests that adolescents who experience peer rejection seek emotional compensation through social media, but that this escapism heightens their sensitivity and defensive suspicion of offline relationships [29]. This finding provides a new perspective on the psychological impact of digital behaviors, highlighting how overuse of social media can exacerbate users’ suspicions.

### The protective and paradoxical role of sense of meaning in life

The mediation analysis yielded theoretically groundbreaking findings that confirm Hypothesis 2: a sense of meaning in life was identified as an inconsistent mediator exhibiting an “erosion-reinforcement” dual mechanism. Specifically, while social suspicion significantly erodes a sense of meaning in life (*β* = -0.175,  *P*< 0.001) – aligning with conventional theories – counterintuitively, the higher sense of meaning in life levels positively predicted social media addiction (*β* = 0.057,  *P*= 0.014), resulting in an overall negative indirect effect (-0.010, 95% CI [-0.019, -0.003]). This paradoxical pattern (Hypothesis 2 supported) suggests that a sense of meaning in life simultaneously manifests protective and risk-enhancing properties: it acts as a psychological buffer against social suspicion, yet inadvertently drives individuals to seek goal attainment and social validation through social media, thereby transforming adaptive strategies into addictive behaviors.

These findings extend the traditional view of the sense of meaning in life as a unidimensional protective factor [23] by revealing its paradoxical role in digital contexts. First, while prior research consistently frames the sense of meaning in life as a solely protective psychological factor [34,35], our mediation analysis demonstrates a functional reversal: social suspicion erodes the sense of meaning in life (*β* = −0.175,  *P*< 0.001), yet preserved meaning inadvertently reinforces social media addiction (*β* = 0.053,  *P*= 0.014). Second, the results highlight self-reinforcing risks within algorithmically structured social environments — meaning-seeking strategies adopted to mitigate social suspicion may evolve into persistent dependency due to platform characteristics, such as algorithm-driven feedback loops [13]. These findings not only expand psychological compensation theory by introducing digital contextuality but also underscore the urgent need for context-sensitive models in media psychology.

### Gender as a moderator

The present study confirms Hypothesis 3 by demonstrating gender differences in the moderating role of gender between social suspicion and sense of meaning in life, i.e., males and females differ in their sense of meaning in life when faced with social suspicion. Specifically, for females, the effect of social suspicion on the sense of meaning in life was more significant. In social science research, gender is often recognized as an important factor that may have an impact on research findings. In terms of suicide risk, men are more likely to engage in violent and lethal behaviors and therefore have a relatively higher risk of suicide [36,37]. However, there is also a gender paradox in the field of suicide research in that women are more inclined to attempt suicide, and men are more inclined to complete it [38,39]. Previous research has shown that males value peer relationships and intimacy more than females, as well as displaying a more complete adult identity [40]. This implies that men are more inclined to display positive attitudes and engage in useful self-reflection during social interactions, thereby promoting social integration and reducing the likelihood of facing social exclusion. However, some researchers have also pointed out that women are more sensitive in perceiving interpersonal stress, and women are often prone to relationship distress due to adverse concerns about relationships [41,42]. Therefore, attention should be paid to gender differences in the mental health status of university students in the process of cultivation, to promote the mental health and personal development of university students, and to provide them with assistance in better adapting to the challenges of society and the future.

In the inverse model, however, we found that men showed stronger predictive effects of social media addiction on social suspicion compared to women. This gender difference may be related to the differences in motivations for social media use and psychological traits between males and females. In terms of motivations for social media use, men may be more inclined to use social media as a tool for presenting themselves and establishing social status, whereas women use it more for maintaining interpersonal relationships and emotional communication [29]. This different motivation for use may lead to men’s social suspicion when they are addicted to social media, as they are more likely to interpret others’ behavior as a threat to their status or image. Second, analyzing from a psychological trait perspective, it has been shown that men typically score higher on the neuroticism personality trait, which is closely associated with emotional states such as anxiety and restlessness [43]. This higher level of neuroticism may make men more prone to negative emotions and suspicion toward others in the context of social media addiction.

The gender differences revealed in this study, while seemingly paradoxical, reflect the multidimensional complexity of socio-psychological mechanisms. On one hand, females exhibit more pronounced fluctuations in their sense of meaning in life when confronted with social suspicion, potentially stemming from their heightened interpersonal sensitivity and stress internalization tendencies [41,44]. Conversely, males demonstrate stronger predictive effects of social media addiction on social suspicion, which may be attributed to their instrumentalized use of social media platforms and neurotic personality traits [36,45]. This apparent paradoxical pattern precisely illustrates that gender functions not as a unidimensional determinant but rather as a dynamic component interacting with psychological characteristics, social role expectations, and media usage patterns [46,47].

From an intervention perspective, these dual disparities necessitate gender-specific mental health support frameworks. For female populations, interventions should emphasize cognitive restructuring training targeting interpersonal hypersensitivity to cultivate more resilient social cognition patterns. For male populations, priority should be given to developing healthy self-presentation strategies and emotion regulation competencies, complemented by digital literacy education to mitigate psychological risks associated with status-driven social media engagement. Future research should further investigate the cultural construction factors and neurobiological underpinnings of these gender disparities, aiming to establish more comprehensive theoretical models that inform precise mental health interventions. Such efforts will ultimately enhance the psychological well-being and social adaptability of university students, equipping them to better navigate evolving societal challenges.

## Limitations and future directions

While this study offers insights into the relationship between social suspicion, social media addiction, and the mediating role of a sense of meaning in life, several limitations warrant consideration and inform future research directions.

### Sample representativeness and generalizability

The study focused on a specific subgroup of college students, which may limit the generalizability of findings to broader populations. Future studies should adopt multi-center sampling strategies across diverse colleges, majors, and demographic backgrounds (e.g., varying age groups, socioeconomic statuses, and cultural contexts). Additionally, longitudinal tracking of participants across different academic years could reveal how developmental stages influence the dynamics between social suspicion, sense of meaning in life, and social media addiction. For instance, social suspicion and its impact on addiction may differ among freshmen versus graduating students due to varying academic and social pressures.

### Self-report biases and measurement validity

The reliance on self-reported measures introduces risks of social desirability bias and recall inaccuracies. To address this, future research could integrate objective behavioral data (e.g., screen-time metrics from smartphones) or physiological indicators (e.g., eye-tracking during social media use) to triangulate findings. Mixed-methods designs incorporating qualitative interviews could also deepen understanding of how students perceive social suspicion and social media addiction, thereby refining measurement tools.

### Narrow scope of the moderated mediation model

The small negative effect size of sense of meaning in life (*β* = -0.010, *P* < 0.01), while statistically significant, suggests its inhibitory role in the social suspicion→social media addiction pathway is modest. This raises questions about its practical significance and whether other unmeasured mediators (e.g., loneliness, self-esteem) or moderators (e.g., coping styles, cultural norms) might amplify or attenuate this effect. Specifically, the weak suppression effect of a sense of meaning in life implies that interventions solely targeting this construct may have a limited impact on reducing social media addiction driven by social suspicion. Future studies should explore alternative pathways, such as testing whether a sense of meaning in life interacts with resilience or social support to produce stronger protective effects. For example, individuals with a high sense of meaning in life and strong social networks might exhibit markedly lower addiction risk, even when social suspicion is present.

In summary, these limitations underscore the complexity of the social suspicion→sense of meaning in life→social media addiction pathway but also chart actionable paths for advancing research. By addressing these gaps, future studies can build more robust models to inform prevention and treatment strategies tailored to college students and beyond.

## Conclusion

**Fig 3:**
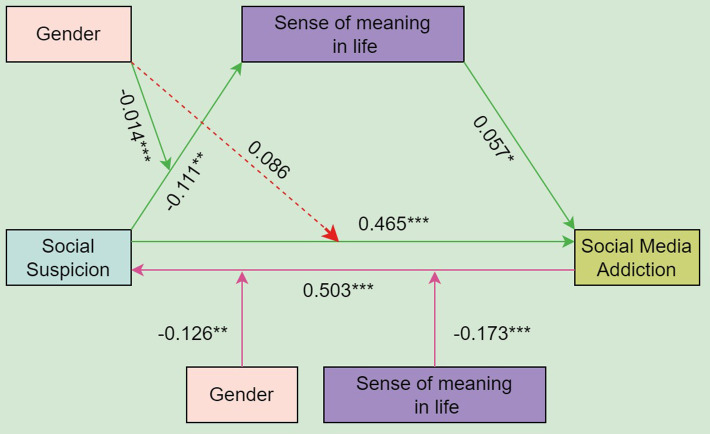
The hypothetical moderated mediation model.

This study elucidates the bidirectional relationship between social suspicion and social media addiction among Chinese college students, mediated by the sense of meaning in life and moderated by gender, as illustrated in Fig 3. Social suspicion directly exacerbates social media addiction while undermining a sense of meaning in life, which paradoxically reinforces addictive behaviors. Gender disparities reveal that females experience stronger mediation through the diminished sense of meaning in life, whereas males exhibit heightened susceptibility to social suspicion when addicted. These findings underscore the need for gender-specific interventions, such as fostering resilient social cognition in females and promoting healthy self-presentation strategies in males. The moderated mediation model critically highlights how psychological and demographic factors dynamically shape digital behaviors, offering actionable pathways to mitigate risks of social media overuse and improve social adaptability in digital contexts, as well as theoretical insights for future research.

## Supporting information

S1 File PROCESS V4.0 Analysis results. (PDF)
