# Supplementary material for: The effect of social suspicion on social media addiction among Chinese college students: A moderated mediation model
Source: PLoS One. 2025 May 21;20(5):e0323474. doi: 10.1371/journal.pone.0323474 (PMC12094756; doi:10.1371/journal.pone.0323474)
Supplement: S1 File — (PDF) [file pone.0323474.s001.pdf]

Run MATRIX procedure:

***************** PROCESS Procedure for SPSS Version 4.0 *****************

Written by Andrew F. Hayes, Ph.D.   www.afhayes.com
Documentation available in Hayes (2022). www.guilford.com/p/hayes3

****

```
Model  : 4
    Y  : 成瘾
    X  : 猜疑
    M  : 生命

Covariates:
 年龄    居住    性别

Sample
Size:  1558
```

****

OUTCOME VARIABLE:
生命

Model Summary

R	R-sq	MSE	F	df1	df2	p
.189	.036	121.667	14.372	4.000	1552.000	.000

Model

	coeff	se	t	p	LLCI	ULCI
constant	60.242	4.305	13.994	.000	51.798	68.686
猜疑	-.153	.022	-7.008	.000	-.196	-.110
年龄	-.475	.233	-2.038	.042	-.931	-.018
居住	-1.452	.705	-2.059	.040	-2.835	-.069
性别	-.146	.561	-.261	.794	-1.246	.954

Standardized coefficients

	coeff
猜疑	-.175
年龄	-.051
居住	-.051
性别	-.007

Covariance matrix of regression parameter estimates:

	constant	猜疑	年龄	居住	性别
constant	18.532	-.011	-.986	-.235	-.221
猜疑	-.011	.000	.000	.000	.001
年龄	-.986	.000	.054	-.008	.004
居住	-.235	.000	-.008	.497	-.019
性别	-.221	.001	.004	-.019	.314

****

OUTCOME VARIABLE:
成瘾

Model Summary

R	R-sq	MSE	F	df1	df2	p
.460	.212	106.975	83.405	5.000	1551.000	.000

Model

	coeff	se	t	p	LLCI	ULCI
constant	39.885	4.284	9.311	.000	31.482	48.287
猜疑	.416	.021	20.000	.000	.375	.456
生命	.055	.024	2.328	.020	.009	.102
年龄	-.640	.219	-2.929	.003	-1.069	-.211
居住	-.144	.662	-.217	.828	-1.443	1.155
性别	.973	.526	1.850	.065	-.059	2.004

Standardized coefficients

	coeff
猜疑	.459
生命	.053
年龄	-.066
居住	-.005
性别	.042

Covariance matrix of regression parameter estimates:

	constant	猜疑	生命	年龄	居住	性别
constant	18.351	-.015	-.034	-.883	-.256	-.199
猜疑	-.015	.000	.000	.000	.000	.001
生命	-.034	.000	.001	.000	.001	.000
年龄	-.883	.000	.000	.048	-.007	.003
居住	-.256	.000	.001	-.007	.438	-.016
性别	-.199	.001	.000	.003	-.016	.277

Test(s) of X by M interaction:

F	df1	df2	p
.037	1.000	1550.000	.847

************************** TOTAL EFFECT MODEL ****************************

OUTCOME VARIABLE:

成瘾

Model Summary

R	R-sq	MSE	F	df1	df2	p
.457	.209	107.280	102.610	4.000	1552.000	.000

Model

	coeff	se	t	p	LLCI	ULCI
constant	43.222	4.042	10.692	.000	35.293	51.151
猜疑	.407	.020	19.871	.000	.367	.447
年龄	-.666	.219	-3.049	.002	-1.095	-.238
居住	-.224	.662	-.339	.735	-1.523	1.075
性别	.965	.527	1.832	.067	-.068	1.997

Standardized coefficients

coeff

猜疑	.450
年龄	-.069
居住	-.008
性别	.041

Covariance matrix of regression parameter estimates:

	constant	猜疑	年龄	居住	性别
constant	16.341	-.010	-.870	-.207	-.194
猜疑	-.010	.000	.000	.000	.001
年龄	-.870	.000	.048	-.007	.003
居住	-.207	.000	-.007	.438	-.016
性别	-.194	.001	.003	-.016	.277

****************** CORRELATIONS BETWEEN MODEL RESIDUALS ******************

	生命	成瘾
生命	1.000	.000
成瘾	.000	1.000

************** TOTAL, DIRECT, AND INDIRECT EFFECTS OF X ON Y **************

Total effect of X on Y

Effect	se	t	p	LLCI	ULCI	c_cs
.450	.023	19.868	.000	.405	.494	.450

Direct effect of X on Y

Effect	se	t	p	LLCI	ULCI	c'_cs
.460	.023	20.026	.000	.415	.505	.460

Indirect effect(s) of X on Y:

	Effect	BootSE	BootLLCI	BootULCI
生命	-.010	.005	-.021	-.001

Completely standardized indirect effect(s) of X on Y:

	Effect	BootSE	BootLLCI	BootULCI
生命	-.010	.005	-.020	-.001

********************** ANALYSIS NOTES AND ERRORS ************************

Level of confidence for all confidence intervals in output:
95.0000

Number of bootstrap samples for percentile bootstrap confidence intervals:
5000

WARNING: Variables names longer than eight characters can produce incorrect output when some variables in the data file have the same first eight characters. Shorter variable names are recommended. By using this output, you are accepting all risk and consequences of interpreting or reporting results that may be incorrect.

------ END MATRIX -----

Run MATRIX procedure:

***************** PROCESS Procedure for SPSS Version 4.0 *****************

Written by Andrew F. Hayes, Ph.D.   www.afhayes.com
Documentation available in Hayes (2022). www.guilford.com/p/hayes3

****
Model : 8
Y : Z成瘾
X : Z猜疑
M : Z生命
W : 性别

Sample
Size: 1558

****
OUTCOME VARIABLE:
Z生命

Model Summary

R	R-sq	MSE	F	df1	df2	p
.186	.035	.967	18.622	3.000	1554.000	.000

Model

	coeff	se	t	p	LLCI	ULCI
constant	.004	.036	.099	.921	-.066	.073
Z猜疑	-.111	.034	-3.242	.001	-.177	-.044
性别	-.014	.050	-2.825	.006	-.112	-.084
Int_1	-.136	.050	-2.721	.007	-.234	-.038

Product terms key:
Int_1   :   Z猜疑   x   性别

Covariance matrix of regression parameter estimates:

	constant	Z猜疑	性别	Int_1
constant	.001	.000	-.001	.000
Z猜疑	.000	.001	.000	-.001
性别	-.001	.000	.002	.000
Int_1	.000	-.001	.000	.003

Test(s) of highest order unconditional interaction(s):

	R2-chng	F	df1	df2	p
X*W	.005	7.403	1.000	1554.000	.007

Focal predict: Z猜疑 (X)
Mod var: 性别   (W)

Conditional effects of the focal predictor at values of the moderator(s):

性别	Effect	se	t	p	LLCI	ULCI
.000	-.111	.034	-3.242	.001	-.177	-.044

```
      1.000      -.247       .037     -6.734       .000      -.319      -.175

Data for visualizing the conditional effect of the focal predictor:
Paste text below into a SPSS syntax window and execute to produce plot.

DATA LIST FREE/
   Z猜疑    性别      Z生命     .
BEGIN DATA.
     -1.000       .000       .114
       .000       .000       .004
      1.000       .000      -.107
     -1.000      1.000       .236
       .000      1.000      -.011
      1.000      1.000      -.257
END DATA.
GRAPH/SCATTERPLOT=
 Z猜疑  WITH     Z生命  BY        性别    .

**************************************************************************
OUTCOME VARIABLE:
 Z成瘾

Model Summary
          R       R-sq        MSE          F        df1        df2          p
       .455       .207       .795    101.526      4.000   1553.000       .000

Model
              coeff         se          t          p       LLCI       ULCI
constant      -.044       .032     -1.356       .175      -.107       .020
Z猜疑        .465       .031     14.980       .000       .404       .525
Z生命        .057       .023      2.465       .014       .012       .102
性别         .086       .045      1.893       .058      -.003       .174
Int_1         -.006       .045      -.124       .902      -.095       .084

Product terms key:
 Int_1    :        Z猜疑   x         性别

Covariance matrix of regression parameter estimates:
           constant     Z猜疑     Z生命      性别       Int_1
constant       .001       .000       .000      -.001       .000
Z猜疑        .000       .001       .000       .000      -.001
Z生命        .000       .000       .001       .000       .000
性别        -.001       .000       .000       .002       .000
Int_1          .000      -.001       .000       .000       .002

Test(s) of X by M interaction:
          F        df1        df2          p
       .002      1.000   1552.000       .963

Test(s) of highest order unconditional interaction(s):
       R2-chng          F        df1        df2          p
X*W       .000       .015      1.000   1553.000       .902
----------
```

```
    Focal predict: Z猜疑  (X)
          Mod var: 性别   (W)

Data for visualizing the conditional effect of the focal predictor:
Paste text below into a SPSS syntax window and execute to produce plot.

DATA LIST FREE/
   Z猜疑     性别      Z成瘾     .
BEGIN DATA.
     -1.000       .000      -.508
       .000       .000      -.044
      1.000       .000       .421
     -1.000      1.000      -.417
       .000      1.000       .042
      1.000      1.000       .501
END DATA.
GRAPH/SCATTERPLOT=
 Z猜疑  WITH     Z成瘾  BY       性别   .

****************** CORRELATIONS BETWEEN MODEL RESIDUALS ******************

           Z生命    Z成瘾
Z生命       1.000       .000
Z成瘾        .000      1.000

****************** DIRECT AND INDIRECT EFFECTS OF X ON Y *****************

Conditional direct effect(s) of X on Y:
     性别     Effect         se          t          p       LLCI       ULCI
       .000       .465       .031     14.980       .000       .404       .525
      1.000       .459       .034     13.623       .000       .393       .525

Conditional indirect effects of X on Y:

INDIRECT EFFECT:
 Z猜疑      ->     Z生命      ->     Z成瘾

     性别     Effect     BootSE   BootLLCI   BootULCI
       .000      -.006       .004      -.015       .000
      1.000      -.014       .007      -.030      -.001

Index of moderated mediation (difference between conditional indirect effects):
           Index     BootSE   BootLLCI   BootULCI
性别      -.008       .005      -.019       .000

 Pairwise contrasts between conditional indirect effects (Effect1 minus Effect2)
    Effect1    Effect2   Contrast     BootSE   BootLLCI   BootULCI
      -.014      -.006      -.008       .005      -.019       .000
---

********************** ANALYSIS NOTES AND ERRORS ************************

Level of confidence for all confidence intervals in output:
```

95.0000

Number of bootstrap samples for percentile bootstrap confidence intervals:
5000

NOTE: Standardized coefficients not available for models with moderators.

------ END MATRIX -----

Run MATRIX procedure:

***************** PROCESS Procedure for SPSS Version 4.0 *****************

Written by Andrew F. Hayes, Ph.D. www.afhayes.com
Documentation available in Hayes (2022). www.guilford.com/p/hayes3

****
Model : 2
Y : Z猜疑
X : Z成瘾
W : 性别
Z : Z生命

Sample
Size: 1558

****
OUTCOME VARIABLE:
Z猜疑

Model Summary

R	R-sq	MSE	F	df1	df2	p
.489	.239	.764	97.291	5.000	1552.000	.000

Model

	coeff	se	t	p	LLCI	ULCI
constant	.064	.032	2.015	.044	.002	.126
Z成瘾	.503	.032	15.890	.000	.441	.565
性别	-.126	.044	-2.834	.005	-.212	-.039
Int_1	-.110	.044	-2.474	.013	-.197	-.023
Z生命	-.173	.022	-7.703	.000	-.216	-.129
Int_2	-.053	.021	-2.567	.010	-.094	-.013

Product terms key:
Int_1 : Z成瘾 x 性别
Int_2 : Z成瘾 x Z生命

Covariance matrix of regression parameter estimates:

	constant	Z成瘾	性别	Int_1	Z生命	Int_2
constant	.001	.000	-.001	.000	.000	.000
Z成瘾	.000	.001	.000	-.001	.000	.000
性别	-.001	.000	.002	.000	.000	.000

```
Int_1          .000      -.001       .000       .002       .000       .000
Z生命        .000       .000       .000       .000       .001       .000
Int_2          .000       .000       .000       .000       .000       .000

Test(s) of highest order unconditional interaction(s):
       R2-chng          F        df1        df2          p
X*W        .003      6.119      1.000   1552.000       .013
X*Z        .003      6.590      1.000   1552.000       .010
BOTH       .006      6.062      2.000   1552.000       .002
----------
    Focal predict: Z成瘾  (X)
          Mod var: 性别   (W)
          Mod var: Z生命  (Z)

Conditional effects of the focal predictor at values of the moderator(s):

     性别     Z生命     Effect         se          t          p       LLCI       ULCI
       .000     -1.000       .556       .038     14.479       .000       .481       .632
       .000       .000       .503       .032     15.890       .000       .441       .565
       .000      1.000       .450       .037     12.078       .000       .377       .523
      1.000     -1.000       .446       .037     12.153       .000       .374       .519
      1.000       .000       .393       .031     12.662       .000       .332       .454
      1.000      1.000       .340       .038      8.967       .000       .266       .414

Data for visualizing the conditional effect of the focal predictor:
Paste text below into a SPSS syntax window and execute to produce plot.

DATA LIST FREE/
   Z成瘾      性别       Z生命      Z猜疑      .
BEGIN DATA.
     -1.000       .000     -1.000      -.320
       .000       .000     -1.000       .236
      1.000       .000     -1.000       .792
     -1.000       .000       .000      -.439
       .000       .000       .000       .064
      1.000       .000       .000       .567
     -1.000       .000      1.000      -.559
       .000       .000      1.000      -.109
      1.000       .000      1.000       .341
     -1.000      1.000     -1.000      -.336
       .000      1.000     -1.000       .111
      1.000      1.000     -1.000       .557
     -1.000      1.000       .000      -.455
       .000      1.000       .000      -.062
      1.000      1.000       .000       .331
     -1.000      1.000      1.000      -.575
       .000      1.000      1.000      -.234
      1.000      1.000      1.000       .106
END DATA.
GRAPH/SCATTERPLOT=
 Z成瘾   WITH      Z猜疑   BY        性别    /PANEL   ROWVAR=  Z生命   .

*********************** ANALYSIS NOTES AND ERRORS ************************
```

Level of confidence for all confidence intervals in output:
  95.0000

Z values in conditional tables are the mean and +/- SD from the mean.

NOTE: Standardized coefficients not available for models with moderators.

------ END MATRIX -----
